# Automatic fitness function selection for compartment model optimization

**DOI:** 10.1186/1471-2202-15-S1-O5

**Published:** 2014-07-21

**Authors:** Timothy Rumbell, Danel Draguljić, Jennifer Luebke, Patrick Hof, Christina M Weaver

**Affiliations:** 1 Fishberg Department of Neuroscience and Friedman Brain Institute, Icahn School of Medicine at Mount Sinai, New York, NY 10029; 2 Department of Mathematics, Franklin and Marshall College, Lancaster, PA, 17604; 3 Department of Anatomy and Neurobiology, Boston University School of Medicine, Boston, MA, 02118

## 

During normal aging, layer 3 pyramidal neurons of the rhesus monkey prefrontal cortex (PFC) exhibit significant morphological changes, as well as higher action potential firing rates *in vitro *[1]. Computational modeling of individual neurons can provide insight into the ionic mechanisms underlying the increased excitability, which are currently unknown. A unique database of electrophysiological recordings and morphologic reconstructions from the same neurons, gathered through whole-cell patch clamp recording, confocal microscopy and 3D digital tracing, constrains the models. Initial modeling of six young and six aged neurons demonstrated that morphological features alone do not account entirely for the electrophysiological changes with aging [2]. It is now necessary to explore the parameter space of passive cable properties and active membrane channel conductances and kinetics, to uncover parameter combinations that reproduce the firing patterns observed in neurons of each age group.

Differential Evolution (DE) is an evolutionary optimization method capable of identifying a population of candidate models throughout parameter space that closely match empirically observed firing patterns. The quality of fit achieved by an optimization is reliant on the ‘fitness functions’ used to measure the accuracy of the model. Previous neuronal compartment modeling studies using parameter optimization have introduced multiple types of fitness measurement [3], but have not described a general method to determine weights for each type. Here we introduce a novel method for automatically establishing weights of minimally correlated fitness functions, and apply it to optimization of models of young and aged PFC neurons. First, a Latin hypercube design (< 1000 points) provides a space-filling sampling of parameter space; the candidate fitness functions are then evaluated at each of these points. Second, clusters of fitness functions that are highly correlated across the hypercube are pruned to leave one representative member. Third, a principal component analysis of the remaining fitness functions across the hypercube identifies a set of fitness functions representing most of the variability in the parameter space, which are selected for use in the optimization. Fourth, weights for each selected fitness function are calculated based on the combination of coefficients for principal components and variance explained by those components. Finally, DE is conducted on the Neuroscience Gateway [4] using this automatically constructed optimization protocol.

We demonstrate the method with a compartment model comprising a simplified pyramidal neuron morphology and three ion channels, optimized to data from representative young and aged neurons. Compared to a manual approach involving iterative generation of fitness functions, our novel method produces better fitting models using a tenth of the computation time. Future work will extend the automatic protocol generation to prioritize which parameters to optimize, a critical step as more ion channels are added to the model to improve fitness. This method will be used to generate morphologically detailed models of 20+ young and aged PFC neurons, predicting which ionic mechanisms underlie age-related physiological changes.
